# Surgeons and Experts’ Perspectives in Concomitant Bariatric Surgery with Cholecystectomy: Bari-Chole Egypt Survey

**DOI:** 10.1007/s11695-026-08907-8

**Published:** 2026-10-05

**Authors:** Mohamed ElKaseer, Mahmoud ElSafty, Hassan El-Masry, Waleed A. Anber, Mohamed S. Elgendy, Hamdy Khaled Sabra, Mohammed Ibrahim El Sayed Henish, Mohamed Tolba, Abd-Elfattah Kalmoush, Mahmoud Ashraf Hussein, Ahmed Abo Elmagd, Khaled Gawdat, Ahmed Abokhozima, Collaborative Bari-Chole Survey Group, Abdelhafez Selim, Abduh Elbanna, Abdelfattah Abozaid, Ahmed Abdelsalam, Ahmed Aboelmagd, Ahmed Alghamery, Ahmed Badawy, Ahmed El Assal, Ahmed Elfeky, Ahmed Fadaly, Ahmed Farid Ahmed, Ahmed Hamza, Ahmed Hassan Mohamed Soliman, Ahmed Hosny Darwish, Ahmed Khalil, Ahmed M. Kandel, Ahmed Mehrez Gad, Ahmed Mohammed Abdelfatah Ali, Ahmed Shibl, Ahmed Taher, Aiman Mandour Ismaeil, Alaa Abbas, Alaa Abdelaty Mahmoud, Alaa Amer, Alaa Badawy, Alaa Sewefy, Aliaa Selim, Amr Madyan, Ashraf Elattar, Ayman Abou Shmeila, Ayman El-Wakeel, Ayman M. Elwan, Emad Abdullah, Eslam Habib, Gamal Maher El Sayed Tofaha, George Ezzat Fahmy Hanna Yacoup, Haitham H. Mohy, Hamdi Elbelkasi, Hamdy Sedky, Hany Armia Balamoun, Hesham A. Elmeligy, Hesham Abdallah, Hosam M. Elghadban, Hosam M. Hamza, Hossam Ramadan Moussa, Husseiny Nabil, Ibrahim Galal, Ibrahim Shalaby, Islam A. El-Sayes, Islam Abdelkhalek, Karim Al Amir, Karim Sabry, Khaled Katari, Mahmoud Abdelnasser Eissa, Mahmoud Eid Sabbak, Mahmoud Sherif, Mina Melad Boshra Yosef, Mohamed Abbas Altaf, Mohamed Abd El Fatah Emam, Mohamed AbdelSalam Newira, Mohamed Abouzeid, Mohamed Ahmed Tolba, Mohamed Alaaeldin Hassan Bakeer, Mohamed Elemawy, Mohamed Elmanakhly, Mohamed Fikry, Mohamed Hany Ashour, Mohamed HossamElDin Hussein Rushdy Zidan, Mohamed Ibrahim Abada, Mohamed Maher Elaraby, Mohamed Mahmoud Abdalgaliel, Mohamed Mokhtar Arafat, Mohamed Ramadan Sobh, Mohamed Shaker, Mohamed Shalamesh, Mohamed Sobhy, Mohamed Sobhy Shaaban Abd El-Kader, Mohammed Abdalmegeed Hamed, Mohammed Ahmed Omar, Mohammed Ammar, Mohammed Arafat Abdel-Maksoud, Mohammed Atef, Mohammed Khalil, Mohammed Mahdy Abdelsalam Sherif, Mohey Elbanna, Mossad Hemida, Mostafa Darwesh, Mostafa Gamal, Mostafa Nagy, Mostafa Refaie Abdelatty Elkeleny, Osama Fathy Almezaien, Osama Osman Khalil, Osama Taha, Reda Fawzy Ali, Sayed Basiony, Sherif Zeidan, Tamer Mohamed Nabil, Tamer Nabil Abdelbaki, Tarek Abouzeid, Waleed Allam, Zahraa Futooh

**Affiliations:** 1 General Surgery Department, Faculty of Medicine, Al-Azhar University, Cairo, Egypt; 2 Faculty of Medicine, Tanta University, Tanta, Egypt; 3 Faculty of Medicine, Alexandria University, Alexandria, Egypt; 4 Department of General Surgery, North Devon District Hospital, Royal Devon University Healthcare NHS Foundation Trust, Barnstaple, Barnstaple, Devon EX31 4JB UK; 5 Kafrelsheikh University, Kafrelsheikh, Egypt; 6 Faculty of Medicine, Capital University, Cairo, Egypt; 7 Department of Surgery, Ain-Shams School of Medicine, Heliopolis, Cairo, Egypt; 8 MIAX Research Lab, Alexandria, Egypt

**Keywords:** Concomitant cholecystectomy, Metabolic bariatric surgery, National survey, Standard practice, Prophylactic cholecystectomy

## Abstract

**Introduction:**

The management of gallbladder disease during metabolic and bariatric surgery (MBS) remains controversial. Cholelithiasis is common in patients with obesity, yet the approach to concomitant or prophylactic cholecystectomy varies, with limited data from Egypt. This study aimed to assess Egyptian bariatric surgeons’ practices, attitudes, and decision-making regarding cholecystectomy during MBS.

**Methods:**

A national cross-sectional survey was distributed to bariatric surgeons in Egypt, collecting information on demographics, surgical experience, preoperative screening, intraoperative decision-making, postoperative management, and prophylactic practices. The questionnaire included structured, closed-ended questions. Data were analyzed descriptively and compared between IFSO members and non-members using chi-square or Fisher’s exact tests, with p < 0.05 considered significant.

**Results:**

A total of 117 surgeons participated, predominantly male (99%), with 16.2% IFSO members. Bariatric surgery was concentrated in Cairo (42%) and Alexandria (26%). Most surgeons (97%) screened gallstones preoperatively using abdominal ultrasound. Concomitant cholecystectomy was preferred for documented gallstones by 78%, while 82% never performed prophylactic cholecystectomy in patients with normal gallbladders. The majority performed the bariatric procedure first (82%) in reverse Trendelenburg position (88%), minimizing additional ports. Routine intraoperative cholangiography was rare (1.7%). Postoperative management largely mirrored isolated bariatric surgery, although antibiotic and drain practices differed between groups. Surgeons generally perceived combined procedures as safe, with 76% reporting no increase in complications and 89% reporting similar length of stay.

**Conclusion:**

Egyptian bariatric surgeons consistently screen for gallstones and favor concomitant cholecystectomy for documented stones, while largely avoiding prophylactic removal of normal gallbladders. Variation exists in intraoperative management and postoperative care. These findings highlight areas for standardization and support the development of a national pathway and multicenter registry to optimize gallbladder management in MBS.

## Introduction

Metabolic and bariatric surgery (MBS) offers effective and sustained treatment of clinically severe obesity, diabetes mellitus, and other associated comorbidities. Sleeve gastrectomy (SG) and Roux-en-Y gastric bypass (RYGB) have favorable safety profiles across different age groups and remain the most commonly performed MBS procedures in the United States, representing nearly 80% of annual MBS cases nationwide [1–3]. Cholelithiasis can be a particularly challenging postoperative complication for patients with MBS, as the anatomical alterations created by MBS procedures may introduce additional technical complexity in subsequent management [4]. As a result, the management of the gallbladder in patients undergoing bariatric surgery remains a clinically relevant issue in modern metabolic and bariatric surgical practice.

Gallstone disease represents a common clinical condition worldwide occurring in approximately 10–20% of the general population and are nearly five times more common among individuals with obesity compared with those of normal body weight [5–7]. Despite this high prevalence, there is no universal consensus regarding the optimal management of the gallbladder during bariatric surgery. Surgical approaches vary widely, ranging from routine concomitant cholecystectomy to selective removal based on symptoms or imaging findings, as well as conservative strategies with postoperative surveillance. Previous studies have shown that the need for cholecystectomy after bariatric procedures such as LAGB, RYGB, and SG ranges from 6.5% to 10.1%, highlighting the importance of appropriate preoperative counseling and consideration of biliary prophylaxis [8].

A 7-year registry study of over 1 million patients demonstrated that concomitant laparoscopic cholecystectomy during bariatric surgery has a favorable safety profile with no increase in perioperative mortality after risk adjustment [9]. Nevertheless, other evidence suggests that concomitant cholecystectomy may increase operative time and postoperative complications, particularly anastomotic-related events, supporting a more selective approach limited to high-risk or symptomatic patients [10]. In the Egyptian context, variations in training pathways, healthcare infrastructure, and surgical volume may further influence clinical decision-making, underscoring the need to understand current practice patterns better. Therefore, our national survey aimed to evaluate the current practices, attitudes, and decision-making of bariatric surgeons in Egypt regarding cholecystectomy during bariatric surgery.

## Methods

### Study Design and Setting

This cross-sectional study was conducted using a national online survey distributed among bariatric surgeons practicing in Egypt. The study aimed to evaluate current surgical practices and decision-making patterns regarding cholecystectomy performed during bariatric surgery. The survey assessed surgeons’ preferred management strategies for symptomatic and asymptomatic gallstones, explored factors influencing intraoperative decision-making, and examined surgeons’ perspectives regarding current evidence and guidelines to support the development of standardized, evidence-based recommendations.

### Target Population and Sampling Strategy

The target population included bariatric surgeons practicing in Egypt. Surgeons affiliated with national bariatric surgery societies and those actively performing bariatric procedures were considered eligible to participate. Participants were recruited using a convenience sampling approach. Invitations were sent to surgeons registered in the national bariatric surgery forum and other professional networks. Multiple reminders were circulated, the study was promoted at national bariatric meetings and professional gatherings, and distribution was reinforced via social media platforms widely used by bariatric surgeons in Egypt, to maximize reach and encourage participation. Because the total number of actively practicing bariatric surgeons in Egypt is not available from a verified national registry, a formal response rate cannot be calculated.

### Survey Instrument Development

Data were collected using a structured self-administered questionnaire developed in English. The questionnaire was designed after reviewing the literature related to gallstone management in bariatric surgery and after consultation with bariatric surgery experts. The survey consisted exclusively of closed-ended questions with predefined answer choices, allowing the collection of standardized quantitative data on practice patterns, perioperative management, and decision-making strategies among bariatric surgeons. The structured format facilitated uniform responses and enabled statistical comparison between different surgeon groups.

### Survey Structure

The questionnaire was organized into several thematic sections to capture clinical practice patterns comprehensively. The demographic section collected information on the surgeon’s gender, city of practice, years of surgical experience, primary surgical specialty, annual bariatric surgery volume, annual combined bariatric and cholecystectomy volume, and whether the surgeon held an active membership in the International Federation for Surgery and Other Therapies for Obesity (IFSO). The preoperative practice section explored whether surgeons routinely screened bariatric patients for gallstones, the screening methods used, such as abdominal ultrasound or other imaging modalities, the management approach when gallstones were detected preoperatively, decisions regarding removal of the gallbladder in asymptomatic patients, and the use of ursodeoxycholic acid for postoperative gallstone prophylaxis.

The intraoperative practice section evaluated the order of procedures when concomitant surgery was performed, preferred patient positioning, trocar placement, and the use of additional ports, the surgeon responsible for performing the cholecystectomy, use of intraoperative cholangiography, techniques used to achieve the critical view of safety, and preferred energy devices such as LigaSure or Harmonic scalpel.

The judgment and decision-making section examined technical preferences and decision-making factors including the site of gallbladder retrieval, the use of drains in the subhepatic area or along the bariatric suture line, factors guiding the decision to perform concomitant cholecystectomy, perceived impact on operative time and complication rates, most concerning complications, management strategies in revisional bariatric surgery, thresholds for staging procedures instead of performing them simultaneously, documentation of the critical view of safety, and the presence of institutional protocols governing cholecystectomy during bariatric surgery.

The postoperative practice section assessed postoperative management including feeding protocols, use and duration of postoperative antibiotics, timing of drain removal, use of routine imaging for postoperative complications, thromboprophylaxis adjustments, commonly encountered complications, length of hospital stay and readmission rates, follow-up practices for biliary complications, existence of institutional registries, postoperative pain levels and analgesia protocols, timing of ambulation and return to normal activity, and the perceived impact on long-term bariatric outcomes. Participation in multicenter registries or collaborative studies was also assessed.

The prophylactic cholecystectomy practice and belief section assessed surgeons’ practices and attitudes toward performing prophylactic cholecystectomy in patients without gallstones, as well as the clinical considerations guiding these decisions. It also explored the sources influencing surgeons’ practice, including guidelines such as those from the American Society for Metabolic and Bariatric Surgery (ASMBS) and IFSO, along with evidence from the literature, personal clinical experience, and recommendations from national surgical societies.

### Survey Distribution and Data Collection

The questionnaire was hosted on Google Forms™, which was used as the online survey platform to facilitate data collection and ensure confidentiality. The survey link was distributed through the national bariatric surgery forum, professional mailing lists, and social media platforms commonly used by bariatric surgeons. The study was also promoted during national bariatric meetings and professional gatherings when applicable. Participation was voluntary, and informed consent was obtained electronically before completion of the questionnaire.

### Statistical Analysis Plan

Data were exported from the survey platform and analyzed using RStudio software version 4.4.3. Categorical variables were summarized as frequencies and percentages. Comparative analyses were conducted to explore differences in practice patterns between IFSO and non-IFSO members. Associations between categorical variables were assessed using Pearson’s chi-square test or Fisher’s exact test when appropriate. A two-sided p-value less than 0.05 was considered statistically significant.

## Results

**Fig. 1:**
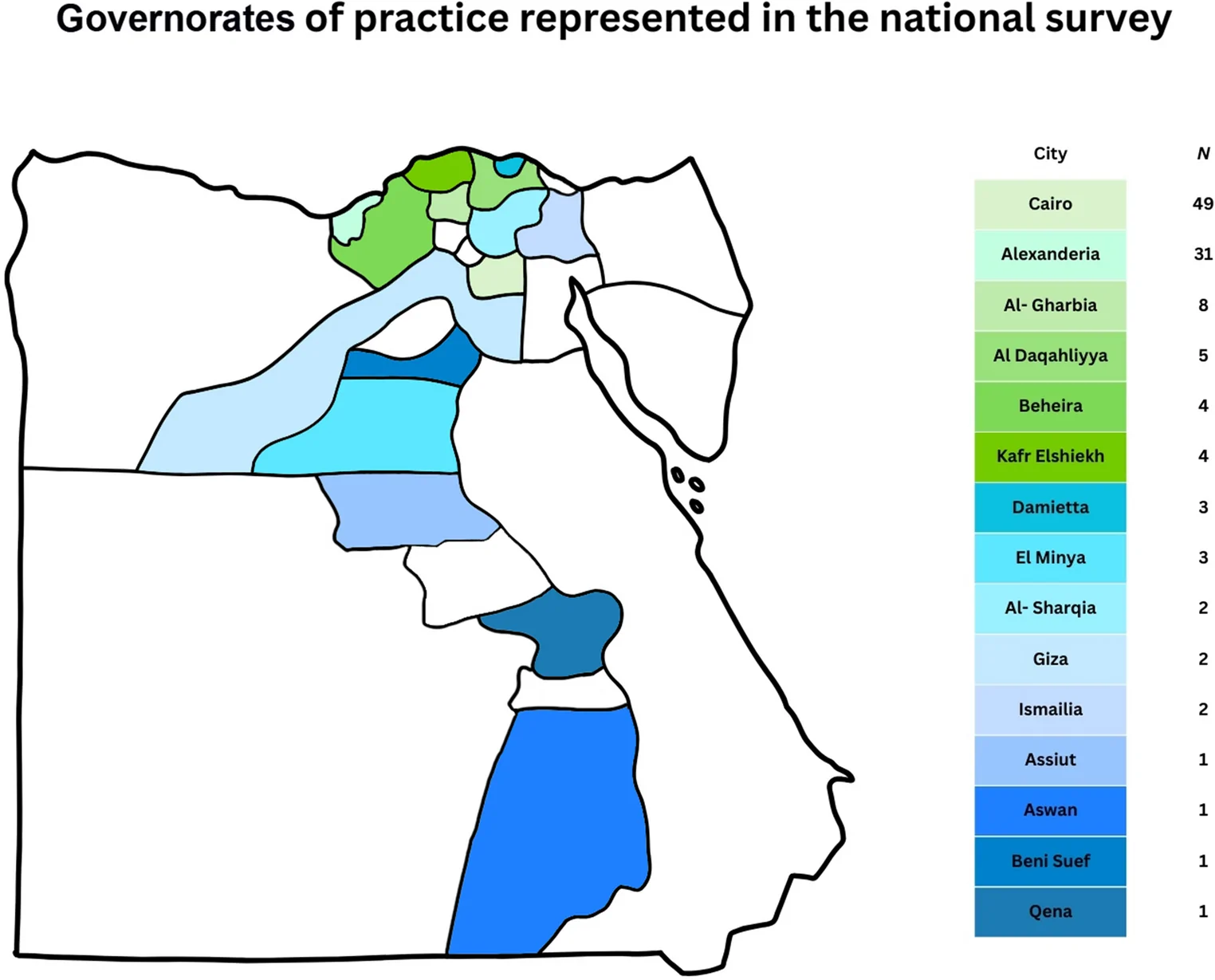
Map showing the city of practice distribution all over Egypt

A total of 117 bariatric and metabolic surgeons were included in this survey. Of them, 99% were male. Nineteen surgeons were registered members of IFSO, representing 16.24% of the cohort, while the remaining participants (83.76%) were non-members. Surgical practice was largely centralized, with 68% of surgeons practicing in Cairo (42%) and Alexandria (26%), while a smaller proportion of surgeons are distributed across other governorates in Upper Egypt and the Delta region (Fig. 1).

Most participants reported extensive surgical experience, with nearly 90% having more than 10 years of practice and approximately one-third exceeding 20 years of experience (28%). This level of experience was reflected in procedural volume, as 47% of surgeons reported performing more than 100 bariatric procedures annually, while nearly 29% reported exceeding 200 cases per year.

Among the surveyed surgeons, 54% identified themselves as bariatric and metabolic surgeons exclusively, whereas 41 surgeons were primarily general surgeons who perform bariatric surgery as part of their general practice. A small proportion of the cohort consisted of hepatobiliary surgeons who occasionally perform bariatric procedures (6%), while 6 surgeons did not specify their primary subspecialty (Table 1).

**Table 1:** Demographics and practice characteristics among the surveyed surgeons

Variables	Overall^a^	IFSO^a^ Members (*N* = 117)		*p*-value^b^
		No^a^ (*N* = 98)	Yes^a^ (*N* = 19)	
Gender				0.2
Female	1 (0.9%)	0 (0%)	1 (5.3%)	
Male	116 (99%)	98 (100%)	18 (95%)	
Years of surgical experience				> 0.99
0–5	1 (0.9%)	1 (1.0%)	0 (0%)	
6–10	11 (9.4%)	10 (10%)	1 (5.3%)	
11–20	72 (62%)	60 (61%)	12 (63%)	
> 20	33 (28%)	27 (28%)	6 (32%)	
Main surgical specialty				0.022
Bariatric	63 (54%)	47 (48%)	16 (84%)	
General	41 (35%)	39 (40%)	2 (11%)	
Hepatobiliary	7 (6.0%)	7 (7.1%)	0 (0%)	
Other	6 (5.1%)	5 (5.1%)	1 (5.3%)	
Average annual bariatric surgery				0.2
< 50	23 (20%)	21 (21%)	2 (11%)	
50–100	39 (33%)	35 (36%)	4 (21%)	
101–200	21 (18%)	17 (17%)	4 (21%)	
> 200	34 (29%)	25 (26%)	9 (47%)	
Average annual combined surgery				0.13
< 50	90 (77%)	79 (81%)	11 (58%)	
50–100	16 (14%)	11 (11%)	5 (26%)	
101–200	8 (6.8%)	6 (6.1%)	2 (11%)	
> 200	3 (2.6%)	2 (2.0%)	1 (5.3%)	
Institution type				0.1
Government hospital	93 (79%)	81 (83%)	12 (63%)	
Private centre	13 (11%)	9 (9.2%)	4 (21%)	
University hospital	11 (9.4%)	8 (8.2%)	3 (16%)	
Governmental + private centre	33 (28%)	23 (23%)	10 (53%)	

^a^n (%)

^b^Fisher’s exact test; Pearson’s chi-square test

**Fig. 2:**
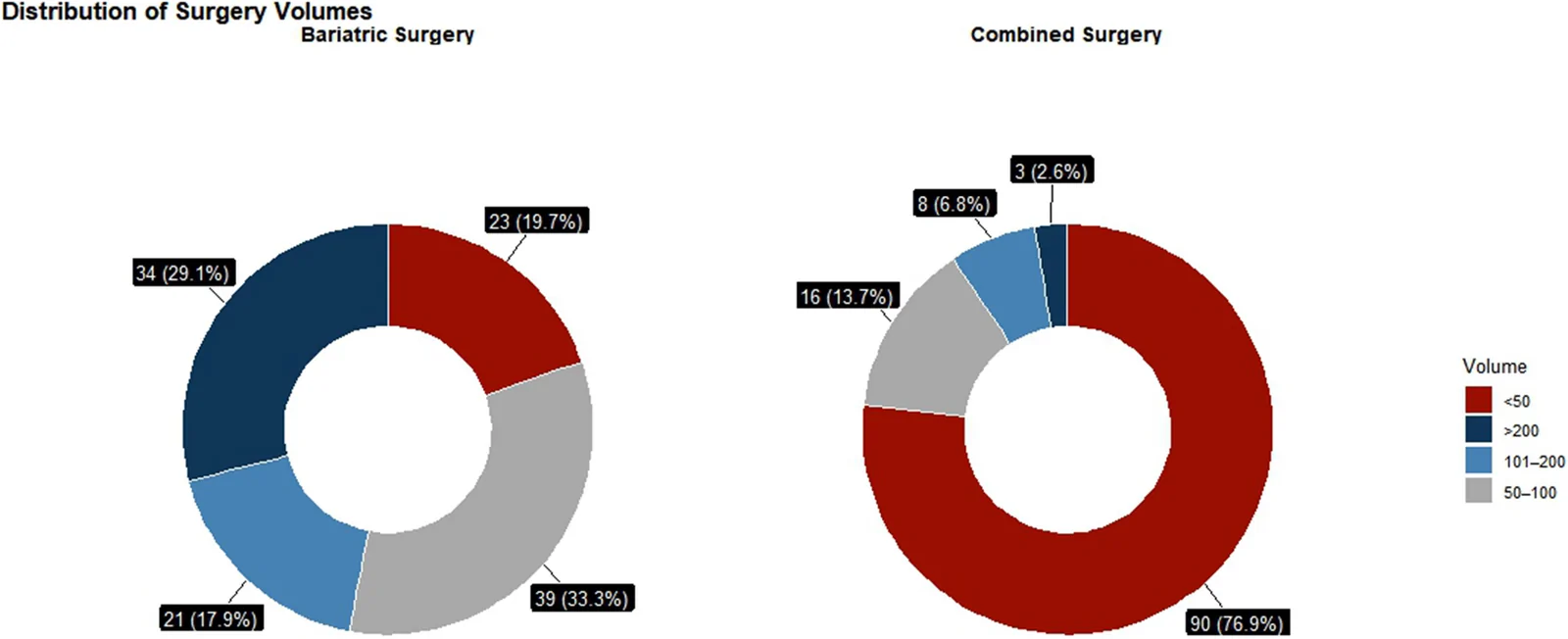
Metabolic bariatric surgery and combined cholecystectomy volume distribution

A notable discrepancy was observed between the annual volume of bariatric surgeries and that of combined bariatric–cholecystectomy procedures. Although 29% of surgeons reported performing more than 200 bariatric operations annually, only 2.6% reported performing a similar volume of combined procedures, with the majority (77%) performing fewer than 50 combined procedures per year (Fig. 2).

### Surgeons’ Screening Practices and Management Preferences for Gallstones in Bariatric Surgery

Abdominal ultrasound was almost universally utilized as the screening modality for gallstones (99%), and 97% of surgeons reported routinely performing preoperative screening. Regarding the management of gallstones detected during preoperative evaluation, surgeons generally favored immediate surgical management.

**Fig. 3:**
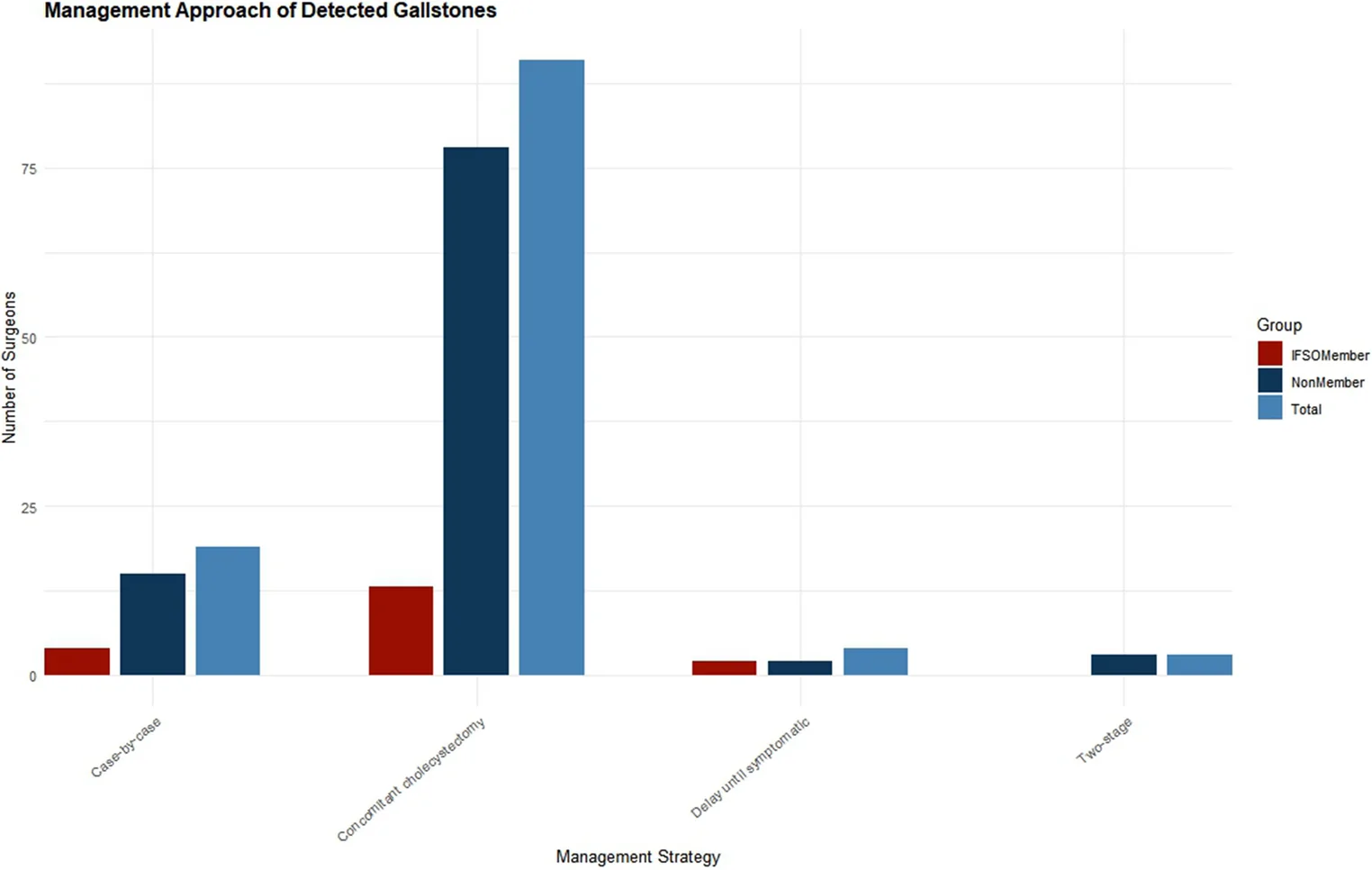
Management approach of detected gallstones before intended metabolic bariatric surgery among surveyed surgeons

Concomitant cholecystectomy performed during the bariatric procedure was the preferred strategy for 78% of surgeons overall, with 68% of IFSO members and 80% of non-members adopting this approach, without a statistically significant difference between the groups (*p* = 0.2) (Table 2). The primary factor guiding the decision to perform a concomitant cholecystectomy was the presence of gallstones themselves (77%), while biliary colic accounted for only 2.3% of decisions (Table 3). Only one surgeon reported making individualized decisions based on patient preference, whereas a minority opted for expectant management and intervened only if symptoms developed postoperatively (3.4%). Performing the procedures in two separate operative sessions was the least commonly adopted strategy (Fig. 3) (Table 2).

**Table 2:** Preoperative assessment and practices for gallstones in bariatric patients

Variables	Overall^a^	IFSO^a^ Members (*N* = 117)		*p*-value^b^
		No^a^ (*N* = 98)	Yes^a^ (*N* = 19)	
Using an abdominal ultrasound for screening				
Yes	116 (99%)	97 (99%)	19 (100%)	> 0.99
Screening for gallstones before bariatric surgeries				
Yes	113 (97%)	97 (99%)	18 (95%)	0.4
Selectively	3 (2.6%)	2 (2.0%)	1 (5.3%)	
Management approach of detected gallstones				0.2
Case-by-case	19 (16%)	15 (15%)	4 (21%)	
Concomitant cholecystectomy (same setting with the bariatric procedure)	91 (78%)	78 (80%)	13 (68%)	
Delay until symptomatic	4 (3.4%)	2 (2.0%)	2 (11%)	
Two-stage	3 (2.6%)	3 (3.1%)	0 (0%)	
Use ursodeoxycholic acid postoperatively for gallstone prophylaxis				0.023
Yes routinely	36 (31%)	25 (26%)	11 (58%)	
Selectively	30 (26%)	26 (27%)	4 (21%)	
No	51 (44%)	47 (48%)	4 (21%)	

^a^n (%)

^b^Fisher’s exact test; Pearson’s chi-square test

**Table 3:** The practice of concomitant cholecystectomy among the surveyed surgeons

Variables	Overall^1^	IFSO^a^ Members (*N* = 87)		*p*-value^b^
		No^a^ (*N* = 75)	Yes^a^ (*N* = 12)	
Factors guide your decision to perform a concomitant cholecystectomy				0.2
Biliary colic	2 (2.3%)	1 (1.3%)	1 (8.3%)	
Comorbidity	2 (2.3%)	2 (2.7%)	0 (0%)	
Gallstones	77 (89%)	67 (89%)	10 (83%)	
Patient preference	1 (1.1%)	1 (1.3%)	0 (0%)	
Type of bariatric procedure	5 (5.7%)	4 (5.3%)	0 (0%)	
Suture line of bariatric procedure draining if a cholecystectomy is done				0.5
Always	44 (51%)	36 (48%)	8 (67%)	
Selectively	34 (39%)	31 (41%)	3 (25%)	
Never	9 (10%)	8 (11%)	1 (8.3%)	
Operative time is significantly increased with concomitant cholecystectomy				0.3
Depending on experience	16 (18%)	16 (21%)	0 (0%)	
Minimally	32 (37%)	27 (36%)	5 (42%)	
Yes	14 (16%)	12 (16%)	2 (17%)	
No	25 (29%)	20 (27%)	5 (42%)	
Concomitant cholecystectomy increases complication rates				0.091
Depending on the procedure	25 (29%)	22 (29%)	3 (25%)	
Yes	7 (8.0%)	4 (5.3%)	3 (25%)	
No	55 (63%)	49 (65%)	6 (50%)	
Performing concomitant cholecystectomy in revisional bariatric cases				0.015
Yes	33 (38%)	24 (32%)	9 (75%)	
No	13 (15%)	13 (17%)	0 (0%)	
Selectively	41 (47%)	38 (51%)	3 (25%)	
What is your threshold for staging the surgeries instead of combining them?				0.084
Difficult anatomy	45 (52%)	41 (55%)	4 (33%)	
High BMI	13 (15%)	9 (12%)	4 (33%)	
Long operative time	17 (20%)	16 (21%)	1 (8.3%)	
Previous surgery	7 (8.0%)	5 (6.7%)	2 (17%)	
Others	4 (5%)	3 (4%)	1 (8.3%)	
No staging	1 (1.1%)	1 (1.3%)	0 (0%)	
Routine documentation of the critical view with photo or video				0.9
Yes	63 (72%)	53 (71%)	10 (83%)	
Sometimes	14 (16%)	13 (17%)	1 (8.3%)	
No	10 (11%)	9 (12%)	1 (8.3%)	
Drainage of the subhepatic area after combined surgery				
Always	39 (45%)	35 (47%)	4 (33%)	0.066
Selectively	42 (48%)	37 (49%)	5 (42%)	
Never	6 (6.9%)	3 (4.0%)	3 (25%)	
Having institutional protocols regarding cholecystectomy during bariatric surgery				0.3
Yes	32 (37%)	26 (35%)	6 (50%)	
NA	55 (63%)	49 (65%)	6 (50%)	

^a^n (%)

^b^Fisher’s exact test; Pearson’s chi-square test

Drain usage patterns varied across the cohort. The use of subhepatic drains differed significantly between IFSO and non-IFSO members (*p* = 0.037), with 44% of non-members routinely using drains, while 26% of IFSO members reported never using them. Overall, 49% of surgeons adopted a selective approach (Table 4). In contrast, bariatric suture line drainage demonstrated greater consensus, with 50% of surgeons routinely placing a drain regardless of the cholecystectomy, and no statistically significant difference between groups (*p* = 0.4).

The use of ursodeoxycholic acid (UDCA) for postoperative gallstone prophylaxis demonstrated considerable variability among surgeons, with a statistically significant difference between IFSO members and non-members (*p* = 0.023). Overall, 31% of surgeons reported routine UDCA use, whereas 26% used it selectively, and 44% did not prescribe UDCA for prophylaxis. Routine use was considerably more common among IFSO members (58%), while 21% of them reported never using the medication.

Surgeons’ Perspectives on Technical Aspects of Combined Procedures.

Most surgeons (82%) preferred the bariatric procedure first, followed by cholecystectomy, while 18% preferred the reverse sequence. The reverse Trendelenburg position was the predominant patient positioning technique (88%), while 12% used the American position, typically applied for standard cholecystectomy. Notably, all surveyed surgeons reported that the same bariatric surgeon performed both components of the procedure.

Most surgeons attempted to minimize the number of trocar sites. Nearly half (48%) performed the cholecystectomy without adding additional ports, and 45% required only one additional port, whereas 6.8% required two extra ports (Table 4). Similarly, 85% of surgeons retrieved the gallbladder through an existing bariatric port, with alternative extraction sites such as the umbilical port (8.5%) or epigastric port (2.6%) used less frequently (Table 4).

**Table 4:** Stated preferences toward concomitant cholecystectomy among the surveyed surgeons

Variables	Overall^a^	IFSO^a^ Members (*N* = 117)		p-value^b^
		No^a^ (*N* = 98)	Yes^a^ (*N* = 19)	
When both procedures are done together, which do you start with?				0.5
Bariatric procedure	96 (82%)	79 (81%)	17 (89%)	
Cholecystectomy	21 (18%)	19 (19%)	2 (11%)	
Preferred patient position				0.054
American position (as cholecystectomy)	13 (11%)	13 (13%)	0 (0%)	
Reverse Trendelenburg	103 (88%)	85 (87%)	18 (95%)	
Semi setting with semi flexed hip and semi flexed knee	1 (0.9%)	0 (0%)	1 (5.3%)	
Who usually performs the cholecystectomy part?				NA
The same bariatric surgeon (performs both the bariatric and cholecystectomy)	117 (100%)	98 (100%)	19 (100%)	
Number of additional ports				0.7
0	56 (48%)	48 (49%)	8 (42%)	
1	53 (45%)	44 (45%)	9 (47%)	
2	8 (6.8%)	6 (6.1%)	2 (11%)	
Routinely perform intraoperative cholangiography				0.6
Yes	2 (1.7%)	2 (2.0%)	0 (0%)	
No	99 (85%)	81 (83%)	18 (95%)	
Selectively	16 (14%)	15 (15%)	1 (5.3%)	
Achieving the critical view of safety				0.2
Lateral approach with curtains down technique	1 (0.9%)	0 (0%)	1 (5.3%)	
Partial proximal approach	1 (0.9%)	1 (1.0%)	0 (0%)	
Standard Calot	112 (96%)	95 (97%)	17 (89%)	
Standard CVS, calot’s is only present in 28% of patients	1 (0.9%)	1 (1.0%)	0 (0%)	
Top-down	2 (1.7%)	1 (1.0%)	1 (5.3%)	
Use of energy devices (LigaSure™, Harmonic™, etc.)				0.3
Always	41 (35%)	33 (34%)	8 (42%)	
Sometimes	73 (62%)	63 (64%)	10 (53%)	
Never	3 (2.6%)	2 (2.0%)	1 (5.3%)	
Gallbladder retrieval site				0.8
Another port from the sleeved stomach extraction site	1 (0.9%)	1 (1.0%)	0 (0%)	
Epigastric	3 (2.6%)	3 (3.0%)	0 (0%)	
Same port	100 (85%)	83 (85%)	17 (89%)	
Separate	3 (2.6%)	2 (2.0%)	1 (5.3%)	
Umbilical	10 (8.5%)	9 (9.2%)	1 (5.3%)	

^a^n (%)

^b^Fisher’s exact test; Pearson’s chi-square test

A strong consensus was observed regarding the dissection technique. Standard Calot triangle dissection to obtain the critical view of safety was used by 96% of surgeons, whereas alternative techniques such as the top-down approach or partial proximal approach were rarely adopted. Routine intraoperative cholangiography (IOC) was uncommon; 1.7% of surgeons performed IOC routinely, 14% used it selectively, while the remaining 84.3% did not perform IOC (Table 3).

### Concomitant Cholecystectomy Practice

Among surgeons performing the concomitant cholecystectomy (78%), decisions to perform concomitant cholecystectomy were predominantly driven by the presence of gallstones (89%), while other factors such as biliary colic, comorbidities, patient preference, and type of bariatric procedure were infrequently cited (*p* = 0.2). Suture line drainage during concomitant procedures was variably applied, with 51% reporting that they always perform drainage, 39% performing it selectively, and 10% never using drainage, with no significant intergroup difference (*p* = 0.5).

Regarding operative considerations, 37% of respondents reported that operative time is minimally increased with concomitant cholecystectomy, while 29% stated no increase, 16% considered it dependent on experience, and 18% reported a significant increase, without significant group differences (*p* = 0.3). Similarly, perceptions of complication risk were divided, with 63% reporting no increase in complications, 29% indicating it depends on the procedure, and 8% reporting increased risk (*p* = 0.091).

A significant difference was observed in the performance of concomitant cholecystectomy during revisional bariatric surgery, where IFSO members reported a higher tendency to perform combined procedures compared with non-IFSO members (75% vs. 32% selectively or 17% no, *p* = 0.015).

The threshold for staging procedures instead of combining them was mainly related to difficult anatomy (52%), followed by long operative time (20%), high BMI (15%), and previous surgery (8%), with no significant difference between groups (*p* = 0.084). Routine documentation of the critical view of safety using photo or video was reported by 72% of respondents, while 16% performed it occasionally and 11% did not, with no intergroup difference (*p* = 0.9).

Drainage of the subhepatic space after combined surgery was performed always by 45%, selectively by 48%, and never by 6.9% (*p* = 0.066). Finally, 37% of respondents reported having institutional protocols guiding cholecystectomy during bariatric surgery, while 63% did not report such protocols, with no significant difference between groups (*p* = 0.3).

### Reported Perspectives Towards Postoperative Management

Most surgeons reported maintaining the same recovery protocols used in isolated bariatric surgery. Specifically, 98% used the same feeding protocol, and 99% reported no change in thromboprophylaxis dose or duration. Routine postoperative imaging was uncommon, with 80% of surgeons performing imaging only when clinically indicated.

Antibiotic practices demonstrated notable variation. Overall, 82% of surgeons administered postoperative antibiotics, though this was more common among non-IFSO members (86%) compared with IFSO members (63%) (Table 5). Furthermore, 51% of surgeons continued antibiotics for more than 72 h, primarily driven by non-IFSO members, while 32% of IFSO members preferred a single-dose approach.

**Table 5:** Postoperative management strategies in combined bariatric and cholecystectomy procedures: surgeons’ reported perspectives

Variables	Overall^a^	IFSO^a^ Members (*N* = 117)		p-value^b^
		No^a^ (*N* = 98)	Yes^a^ (*N* = 19)	
Standard postoperative feeding protocol after combined surgery				> 0.99
Clear fluids longer	1 (0.9%)	1 (1.0%)	0 (0%)	
Delayed	1 (0.9%)	1 (1.0%)	0 (0%)	
Same as standard bariatric	115 (98%)	96 (98%)	19 (100%)	
Routine usage of postoperative antibiotics				0.037
Yes for all	96 (82%)	84 (86%)	12 (63%)	
Only high-risk	8 (6.8%)	5 (5.1%)	3 (16%)	
Single dose	9 (7.7%)	7 (7.1%)	2 (11%)	
No	4 (3.4%)	2 (2.0%)	2 (11%)	
Duration of antibiotics				0.022
Single dose	15 (13%)	9 (9.2%)	6 (32%)	
24 h	5 (4.3%)	4 (4.1%)	1 (5.3%)	
48–72 h	37 (32%)	30 (31%)	7 (37%)	
> 72 h	60 (51%)	55 (56%)	5 (26%)	
Time of cholecystectomy drain removal (if used)				0.3
< 24 h	62 (53%)	48 (49%)	14 (74%)	
24–48 h	36 (31%)	33 (34%)	3 (16%)	
>48 h	1 (0.9%)	1 (1.0%)	0 (0%)	
Until output < 30 mL	18 (15%)	16 (16%)	2 (11%)	
Time of bariatric drain removal (if used)				0.024
< 24 h	55 (47%)	40 (41%)	15 (79%)	
24–48 h	33 (28%)	31 (32%)	2 (11%)	
>48 h	10 (8.5%)	10 (10%)	0 (0%)	
Until output < 30 mL	19 (16%)	17 (17%)	2 (11%)	
Routine postoperative imaging for complications				0.6
Yes routinely	11 (9.4%)	9 (9.2%)	2 (11%)	
Only if suspicion	94 (80%)	80 (82%)	14 (74%)	
Never	12 (10%)	9 (9.2%)	3 (16%)	
Modification of thromboprophylaxis dose for combined cases				> 0.99
Extended	1 (0.9%)	1 (1.0%)	0 (0%)	
Same as bariatric	116 (99%)	97 (99%)	19 (100%)	

^a^n (%)

^b^Fisher’s exact test; Pearson’s chi-square test

Drain removal practices also differed between groups. 79% of IFSO members removed the bariatric suture line drain within 24 h, compared with 41% of non-IFSO members. For subhepatic drains, when used, 53% of surgeons removed them within 24 h (Table 5).

### Surgeons’ Perspectives on Outcomes, Recovery, and Follow-Up

The majority considered the combined procedure safe, with 76% reporting no increase in complications and 18% reporting complication rates comparable to isolated bariatric surgery. When complications occurred, port-site infection was the most commonly reported (43%), followed by bleeding (20%) and bile leak (16%). The risk of readmission due to biliary complications was extremely low, with 86% of surgeons reporting rates below 1%.

Recovery following combined surgery was generally comparable to isolated bariatric procedures. 89% of surgeons reported similar hospital length of stay, all surgeons reported similar rates of early ambulation, and 91% indicated that patients returned to normal activity within one week (Table 6). 65% reported post operative pain levels similar to isolated procedures, while 25% believed pain was higher in combined operations.

**Table 6:** Surgeons’ perspectives on outcomes, recovery, and follow-up after combined bariatric and cholecystectomy procedures

Variables	Overall^a^	IFSO^a^ Members (*N* = 117)		*p*-value^b^
		No^a^ (*N* = 98)	Yes^a^ (*N* = 19)	
Observed higher postoperative complications in combined cases				0.12
Yes	7 (6.0%)	4 (4.1%)	3 (16%)	
Comparable	21 (18%)	19 (19%)	2 (11%)	
No	89 (76%)	75 (77%)	14 (74%)	
Most frequently observed complications				0.4
Bile leak	19 (16%)	18 (18%)	1 (5.3%)	
Bleeding	23 (20%)	19 (19%)	4 (21%)	
Ileus	6 (5.1%)	5 (5.1%)	1 (5.3%)	
Port-site infection	50 (43%)	41 (42%)	9 (47%)	
No complications	15 (13%)	13 (13%)	2 (11%)	
Average length of hospital stay after combined surgery				0.2
Same	104 (89%)	87 (89%)	17 (89%)	
+ 1 day	4 (3.4%)	2 (2.0%)	2 (11%)	
+ 2–3 days	7 (6.0%)	7 (7.1%)	0 (0%)	
> 3 days	2 (1.7%)	2 (2.0%)	0 (0%)	
Approximate readmission rate for biliary/abdominal issues				0.5
<1%	101 (86%)	83 (85%)	18 (95%)	
1–5%	15 (13%)	14 (14%)	1 (5.3%)	
6–10%	1 (0.9%)	1 (1.0%)	0 (0%)	
Routine follow-up for biliary complications				0.4
Yes	27 (23%)	22 (22%)	5 (26%)	
Only symptomatic	79 (68%)	68 (69%)	11 (58%)	
No protocol	11 (9.4%)	8 (8.2%)	3 (16%)	
Maintenance of the institutional data/registry on biliary complications				0.9
Yes	60 (51%)	50 (51%)	10 (53%)	
Planned	24 (21%)	21 (21%)	3 (16%)	
No	33 (28%)	27 (28%)	6 (32%)	
Postoperative pain levels compared to isolated bariatric cases				0.3
Higher	29 (25%)	27 (28%)	2 (11%)	
Similar	76 (65%)	61 (62%)	15 (79%)	
Not sure	12 (10%)	10 (10%)	2 (11%)	
Analgesia protocol				0.069
Multimodal	38 (32%)	29 (30%)	9 (47%)	
Regional block	5 (4.3%)	3 (3.1%)	2 (11%)	
Standard	74 (63%)	66 (67%)	8 (42%)	
Early ambulation after combined surgery				
Same as standard	117 (100%)	98 (100%)	19 (100%)	NA
Return to normal activity				> 0.99
1 week (same as bariatric)	107 (91%)	89 (91%)	18 (95%)	
2 weeks	6 (5.1%)	5 (5.1%)	1 (5.3%)	
3–4 weeks	4 (3.4%)	4 (4.1%)	0 (0%)	
Combined surgery affects long-term bariatric outcomes				0.044
Yes	17 (15%)	17 (17%)	0 (0%)	
Maybe	21 (18%)	15 (15%)	6 (32%)	
No	79 (68%)	66 (67%)	13 (68%)	
Would you join a multicentre registry or collaborative study on this topic?	82 (70%)	68 (69%)	14 (74%)	0.7

NA; not applicable

^a^n (%)

^b^Fisher’s exact test; Pearson’s chi-square test

Only 23% of surgeons routinely monitored patients for biliary complications beyond standard bariatric follow-up protocols, while 68% provided additional follow-up only if symptoms developed (Table 6). Additionally, 51% of surgeons maintained institutional registries for biliary complications, and 70% expressed willingness to participate in a multicenter registry or collaborative study.

### Prophylactic Cholecystectomy Practices and Attitudes

**Fig. 4:**
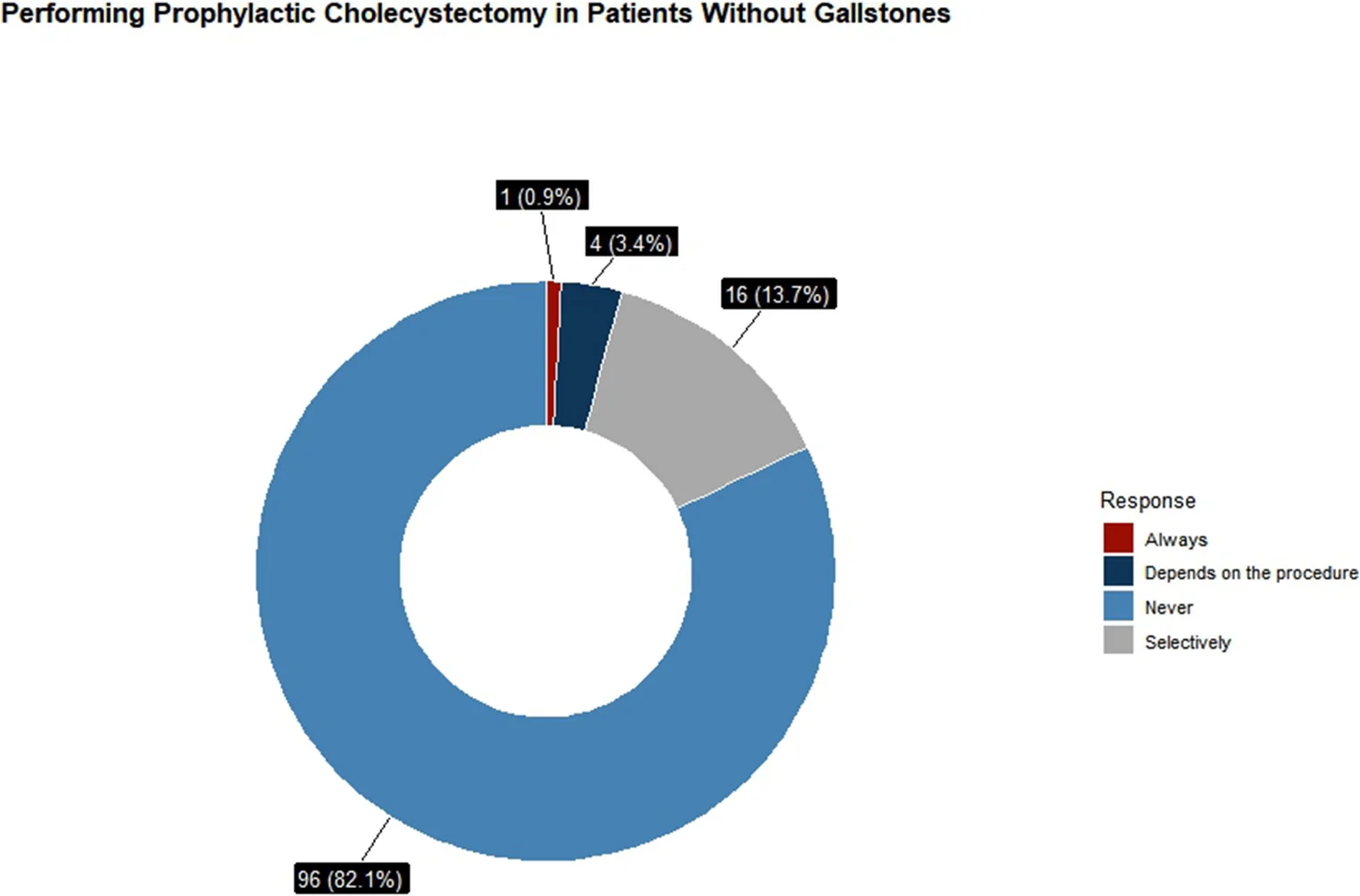
Prophylactic cholecystectomy practice among surveyed surgeons

A strong consensus against this practice was observed, with the majority of surgeons opting not to perform prophylactic cholecystectomy in patients without gallstones, 14% performing it selectively, and only one surgeon reporting routine use (Fig. 4). No statistically significant differences were observed between IFSO members and non-members (*p* = 0.3). Among surgeons who perform prophylactic cholecystectomy, sleeve gastrectomy was the most frequently combined procedure (7.7%), followed by Roux-en-Y gastric bypass (5.1%) and one-anastomosis gastric bypass (3.4%). Because the practice is uncommon, 80% of surgeons reported that questions regarding operative time or morbidity were not applicable. Awareness of formal guidelines was limited; 85% of surgeons reported no knowledge of institutional or national recommendations, and 79% did not cite any guidelines influencing their practice. Among those referencing guidelines, IFSO guidelines were cited by 14%, ASMBS guidelines by 2.6%, and general literature by 1.7% (Table 7).

**Table 7:** Practices and attitudes toward prophylactic cholecystectomy in bariatric surgery

Variables	Overall^a^	IFSO^a^ Members (*N* = 117)		*p*-value^b^
		No^a^ (*N* = 98)	Yes^a^ (*N* = 19)	
Performing prophylactic cholecystectomy in patients without gallstones				0.3
Always	1 (0.9%)	0 (0%)	1 (5.3%)	
Depends on procedure	4 (3.4%)	4 (4.1%)	0 (0%)	
Selectively	16 (14%)	14 (14%)	2 (11%)	
Never	96 (82%)	80 (82%)	16 (84%)	
Most common bariatric procedure combined with prophylactic cholecystectomy				0.8
No prophylactic cholecystectomy	96 (82%)	80 (81%)	16 (84%)	
OAGB	4 (3.4%)	3 (3.1%)	1 (5.3%)	
RYGB	6 (5.1%)	6 (6.1%)	0 (0%)	
SASI	1 (0.9%)	1 (1.0%)	0 (0%)	
Sleeve	9 (7.7%)	7 (7.1%)	2 (11%)	
Does prophylactic cholecystectomy significantly prolong operative time?				0.6
Yes	8 (6.8%)	8 (8.2%)	0 (0%)	
Minimal	9 (7.7%)	7 (7.1%)	2 (11%)	
No	6 (5.1%)	5 (5.1%)	1 (5.3%)	
No prophylactic cholecystectomy	94 (80%)	78 (80%)	16 (84%)	
Increase early morbidity with prophylactic cholecystectomy				> 0.99
Yes	3 (2.6%)	3 (3.1%)	0 (0%)	
Maybe	9 (7.7%)	8 (8.2%)	1 (5.3%)	
No	11 (9.4%)	9 (9.2%)	2 (11%)	
No prophylactic cholecystectomy	94 (80%)	78 (80%)	16 (84%)	
Does prophylactic cholecystectomy reduce long-term biliary issues?				> 0.99
Yes	10 (8.5%)	8 (8.2%)	2 (11%)	
Maybe	8 (6.8%)	7 (7.1%)	1 (5.3%)	
No	2 (1.7%)	2 (2.0%)	0 (0%)	
Unsure	3 (2.6%)	3 (3.1%)	0 (0%)	
No prophylactic cholecystectomy	94 (80%)	78 (80%)	16 (84%)	
Awareness of any institutional/national guidelines on prophylactic cholecystectomy				0.14
Yes	99 (85%)	13 (13%)	1 (5.3%)	
Unsure	99 (85%)	2 (2.0%)	2 (11%)	
No	99 (85%)	83 (85%)	16 (84%)	
Guideline most influencing your practice				0.2
ASMBS	3 (2.6%)	1 (1.0%)	2 (11%)	
IFSO	16 (14%)	15 (15%)	1 (5.3%)	
Literature	2 (1.7%)	2 (2.0%)	0 (0%)	
National society	1 (0.9%)	1 (1.0%)	0 (0%)	
Personal experience	2 (1.7%)	2 (2.0%)	0 (0%)	
Didn’t mention any guidelines	93 (79%)	77 (79%)	16 (84%)	

^a^n (%)

^b^Fisher’s exact test; Pearson’s chi-square test

## Discussion

Based on our national survey of 117 Egyptian bariatric surgeons, the overall strategy for gallbladder management is consistent, but perioperative details vary. Almost all surgeons screen for gallstones (97%), nearly always using ultrasound (99%). When gallstones are detected, most prefer concomitant cholecystectomy (78%), while prophylactic cholecystectomy in patients without stones is largely rejected (82% never). Preventive practice is the main area of disagreement: UDCA is prescribed routinely by 31%, selectively by 26%, and not used by 44%, with routine use differing by IFSO status (26% in IFSO vs. 58% in non-IFSO). Postoperative routines also differ, particularly drain use and antibiotic practice. Despite this variation, most surgeons perceive combined surgery as safe, reporting no increase in complications (76%) and no change in length of stay (89%).

Gallbladder management during bariatric and metabolic surgery is common in daily practice, yet surgeons still face uncertainty because recommendations are not applied uniformly. In this national survey, we used a structured questionnaire to capture contemporary decision-making among Egyptian bariatric surgeons across different practice settings, and we examined patterns by professional society affiliation (IFSO membership). This approach is novel in our context because it provides a clear map of current practice rather than relying on assumptions, single-center experience, or indirect estimates. By defining where practice is consistent and where it is not, the study fills an important local evidence gap and sets the stage for a focused discussion on standardization, guideline uptake, and future research priorities.

Current guidance supports a selective approach to gallbladder management around MBS [11, 12]. Routine prophylactic cholecystectomy for an asymptomatic, normal gallbladder is generally discouraged, while concomitant cholecystectomy is considered reasonable and safe when there is symptomatic gallbladder disease or another clear indication [11, 12]. For bypass-type operations (e.g., RYGB or duodenal switch), experts emphasize that postoperative access to the biliary tree with standard ERCP can be difficult; therefore, surgeons are encouraged to use intraoperative biliary imaging (IOC or an alternative modality when IOC is not feasible) to clarify anatomy and detect ductal stones so they can be managed in the same setting when possible [11, 13]. If concomitant cholecystectomy is not safe, delaying it until early weight loss improves exposure is reasonable.

In this national survey of Egyptian bariatric surgeons, practice reflected a selective but intervention-leaning strategy for gallbladder management. When gallstones were identified preoperatively, most surgeons preferred concomitant cholecystectomy (78%), whereas prophylactic cholecystectomy for a normal gallbladder was strongly rejected (82% never). This pattern is broadly consistent with the ASMBS Foregut Committee review, which discourages routine prophylactic cholecystectomy in asymptomatic patients and considers concomitant cholecystectomy acceptable and safe when symptomatic gallbladder disease is present [12]. Prior work also supports this selective approach as cost-effective [14]. However, the literature remains mixed: several single-center studies report feasibility and safety of concomitant cholecystectomy [15–17]. and highlight the challenges of interval surgery after gastric bypass [18], while larger meta-analyses and registry-based studies suggest higher major complication rates when cholecystectomy is added to MBS [19–21].

At the same time, we observed clear heterogeneity in perioperative adjuncts (UDCA prophylaxis, drainage, antibiotics, and IOC use), and two-thirds of respondents reported no institutional protocol guiding these decisions. These findings matter because biliary events remain a common and sometimes difficult-to-manage problem after MBS, and practice variation often reflects both gaps and system-level differences in resources and follow-up.

In Egypt, the strong consensus against removing a normal gallbladder is consistent with this direction. However, the high preference for concomitant cholecystectomy when stones are detected, where the presence of gallstones outweighs biliary colic as the main driver (75% vs. 8.5%), suggests that many surgeons treat asymptomatic stones as a sufficient indication for removal during MBS. This preference may be influenced by both the evidence base and postoperative access to care. Meta-analytic estimates indicate that de novo gallstones after bariatric surgery are common, while symptomatic disease is lower but still clinically meaningful; Amorim-Cruz et al. reported de novo gallstones in ~ 20.7% and symptomatic gallstone disease in ~ 8.2% over roughly 29 months of follow-up [21].

De Barros et al., using scheduled ultrasound surveillance after SG and RYGB in patients without baseline stones, found a 13.8% incidence that was mostly asymptomatic, with earlier diagnosis after RYGB and an association with MASLD [22]. These figures help explain why prophylactic cholecystectomy in a normal gallbladder is hard to justify, but they do not fully answer what to do with pre-existing stones, which carry a higher likelihood of future cholecystectomy [19].

Several studies support the idea that known gallstones (even asymptomatic) may behave differently in the bariatric population. Xia et al. found that patients with preoperative gallstones were more likely to need later cholecystectomy than those with normal gallbladders, reinforcing a “treat pathology when present” concept [19]. In a multicenter randomized trial of sleeve gastrectomy in patients with asymptomatic gallstones, Habeeb et al. reported that more than half of patients in the SG-only arm (55%) developed symptomatic disease requiring cholecystectomy within two years, most within the first year [16]. This type of signal likely influences surgeon behavior: if gallstones are already present, many surgeons prefer to avoid the risk of early biliary admission and a second operation during the rapid-weight-loss period, especially in systems where follow-up may be fragmented.

At the same time, the evidence helps explain why some surgeons remain cautious about adding cholecystectomy to bariatric surgery. In a meta-analysis of 18 observational studies (105,555 patients), Xia et al. found that concomitant cholecystectomy prolonged operative time by about 21 min (MD 21.06 min) and was associated with a modest increase in overall postoperative complications (OR 1.23), while mortality (OR 1.3) and length of stay (MD 0.11 days) were not significantly different [19]. Similarly, in a Bayesian meta-analysis, Amorim-Cruz et al. reported a high probability of increased major complications with prophylactic concomitant cholecystectomy (OR 1.74; 97% probability of higher major complications) and longer operative time (MD 29.2 min), with no meaningful differences in mortality (OR 0.79) or length of stay (MD − 0.1 days) [21]. In confirmed gallstones, Soares et al. reported higher odds of bleeding, wound, respiratory, and anastomotic complications with concomitant cholecystectomy and advised reserving it for high-risk or symptomatic patients, especially avoiding anastomotic procedures when possible [10].

Against this backdrop, it is notable that Egyptian surgeons articulated clear “stop points” for staging rather than combining, most commonly difficult anatomy (46%), followed by concerns about long operative time (21%) and high BMI (18%). This suggests that, even among surgeons who generally favor concomitant cholecystectomy, case selection remains central and may be acting as the main safety mechanism.

Our technical findings suggest that many surgeons have integrated cholecystectomy into the bariatric workflow in a way they feel is smooth and minimally disruptive. When a combined operation is performed, most respondents start with the bariatric procedure (82%), position the patient in reverse Trendelenburg (88%), and keep any added invasiveness to a minimum (0–1 extra port in 93%, with gallbladder retrieval through the same port in 85%). Consistent with this, most surgeons did not perceive a major impact on operative time (only 21% reported a significant increase), and three-quarters reported no rise in complications (76%). These impressions broadly match reports from large single-center series suggesting that concomitant cholecystectomy is feasible with only modest added time (6). However, survey-based perceptions should still be interpreted carefully in the absence of audited outcomes.

One area where our findings diverge from some recommendations is the sequence of procedures. While most Egyptian surgeons reported performing the bariatric operation first (82%), the ASMBS review notes the rationale for performing cholecystectomy first in certain combined scenarios (particularly with RYGB/BPD‑DS) to mitigate the consequences of a bile duct injury [12]. The practical explanation may be that many combined cases are paired with sleeve gastrectomy (which typically preserves standard biliary access), and surgeons may prioritize completing the metabolic operation before shifting fields.

Nonetheless, the sequencing question is a useful target for a future national pathway: the “best” order likely depends on the bariatric procedure type, the anticipated difficulty of Calot’s triangle, and the team’s capacity to manage a rare but high-impact bile duct injury. Biliary duct evaluation is another important point of discussion. Routine IOC use in our survey was very low (1.7%), with selective use in only 14%. This is relevant because recent SAGES guidance suggests that in patients with bypass-type anatomy undergoing laparoscopic cholecystectomy, surgeons should consider routine intraoperative biliary imaging (IOC preferred, or alternatives when IOC is not feasible), given that standard ERCP may be difficult later and intraoperative imaging allows same-setting intervention if stones are detected; the recommendation is conditional and based on low-quality evidence, and imaging may be omitted when negative preoperative MRCP reliably excludes duct stones [11].

Our survey question addressed “routine IOC” in combined cases broadly rather than specifically in altered anatomy or high-risk ductal stone scenarios, which may partly explain the low rate. Still, the finding highlights a potential gap between evolving recommendations for altered anatomy and day-to-day practice, likely driven by equipment availability, operative flow, and variable confidence with transcystic exploration. UDCA prophylaxis was one of the clearest signals of practice heterogeneity. Only 31% used UDCA routinely, 26% selectively, and 44% not at all, with a significant difference by IFSO membership status. This spread mirrors global uncertainty: in the international expert survey by Kermansaravi et al., 57% prescribed UDCA prophylaxis, most often for 6 months, reflecting broad but not universal acceptance [23]. Mesquita et al. emphasize that UDCA for the early rapid-weight-loss period can substantially reduce stone formation and support a selective strategy that avoids prophylactic cholecystectomy in asymptomatic patients [24].

In Egypt, this split likely reflects real-world barriers such as medication cost, adherence, and follow-up continuity, as well as different views on “medical prevention” versus “one-time surgical prevention” when gallstones are already present [13]. Variation was also clear in perioperative adjuncts that can affect outcomes and resource use. Subhepatic drain practice ranged from “always” (42%) to “never” (9.4%), and antibiotic use was inconsistent: 82% prescribed postoperative antibiotics for all patients, and 51% continued antibiotics beyond 72 h, with significant differences by IFSO membership. In contrast, most surgeons reported no change to the standard bariatric pathway for recovery: feeding was the same in 98% and thromboprophylaxis in 99%, suggesting that many teams treat concomitant cholecystectomy as an added step rather than a reason to alter postoperative care.

The survey also sheds light on follow-up philosophy. Only 23% reported routine biliary follow-up, while 68% followed patients for biliary issues only if symptoms developed. This reactive model likely reinforces the desire to “solve the gallbladder problem” in the index operation when stones are present, particularly because biliary symptoms after bypass procedures can be harder to diagnose and treat. It is encouraging, however, that 51% reported maintaining a registry on biliary complications (and 21% planned one), and 70% expressed willingness to join a multicenter registry. That level of engagement creates a realistic opportunity to move beyond opinion and toward outcome-based national recommendations.

Taken together, the Egyptian practice pattern is broadly concordant with the international expert community and with current SAGES 2025 and ASMBS guidance on the three anchor decisions: routine preoperative screening, concomitant cholecystectomy for documented gallstones, and avoidance of prophylactic excision in a normal gallbladder [25, 26]. The most consequential gaps are in UDCA adoption where Egyptian surgeons trail both the Kermansaravi et al. and the SAGES recommendation to prescribe it routinely through the rapid weight-loss phase when cholecystectomy is deferred and in intraoperative biliary imaging, where fewer than 2% use IOC routinely despite a conditional SAGES recommendation for its use in bypass-type anatomy. Guideline unawareness, reported by 85% of respondents, likely explains both gaps and underscores that the divergence reflects a dissemination failure rather than a substantive disagreement with the evidence [23, 25, 26].

The survey findings, taken together, support three actionable priorities. First, an evidence-informed national decision pathway for gallbladder management in MBS should be developed, anchored by the consensus points identified in this survey: routine preoperative ultrasound screening, concomitant cholecystectomy as the default when gallstones are present, and avoidance of prophylactic cholecystectomy in a normal gallbladder. The pathway should include explicit guidance on procedure sequencing by bariatric procedure type, thresholds for staging rather than combining, UDCA prophylaxis for patients undergoing bypass-type procedures without cholecystectomy, and criteria for intraoperative biliary imaging in altered-anatomy cases. Second, targeted education is needed on IOC and alternative biliary imaging in bypass-type anatomy, where postoperative ERCP may be difficult and the consequences of a missed ductal stone are significant; the gap between current practice (1.7% routine IOC) and evolving SAGES guidance is a concrete and addressable target. Third, a national multicenter outcomes registry should be established, tracking gallstone status at baseline, bariatric procedure type, whether cholecystectomy was performed concurrently or staged, UDCA use, imaging strategy, and hard endpoints (operative time, bile duct injury, anastomotic complications, readmissions, and subsequent biliary interventions). A Delphi consensus process, using the current survey findings to define the domain of variation and areas of uncertainty, would be a logical and high-yield next step toward developing evidence-informed national recommendations for gallbladder management in bariatric surgery in Egypt.

This study has the usual strengths and limitations of a national expert-opinion survey. Its strengths include the comprehensive national scope, the inclusion of highly experienced surgeons with substantial bariatric volumes, and the detailed capture of intraoperative and postoperative decision-making patterns that are rarely described in the region. In addition, the study offers broad scoping across multiple aspects of cholecystectomy in the context of bariatric surgery, with particular emphasis on prophylactic cholecystectomy, a topic that has recently gained significant attention and concern due to its potential implications for operative safety, perioperative outcomes, and long-term patient management [21, 27, 28]. By examining both commonly performed practices and areas of ongoing debate, the survey provides a nuanced view of clinical decision-making, highlighting where consensus exists, where variation persists, and where further evidence and guideline development are most needed.

This study has limitations, mostly due to being a survey research. First, the findings reflect self-reported practice opinions, which may differ from real clinical activity and are vulnerable to recall and social desirability bias. Second, although responses were collected nationally, the sample may not fully represent all bariatric surgeons or practice settings in Egypt, introducing selection and non-response bias and limiting generalizability. Third, a formal response rate cannot be calculated because no verified census of actively practicing bariatric surgeons in Egypt currently exists; the denominator is unknown, and results should be interpreted accordingly. Fourth, subgroup analyses (e.g., IFSO members vs. non-members) included relatively small, unequal groups, which may reduce statistical power and increase the risk of type II error; all such comparisons should be treated as exploratory. Fifth, the survey design is cross-sectional, capturing practice at a single time point; practices may evolve with emerging evidence, availability of resources, and guideline updates. Sixth, we did not capture granular clinical variables (e.g., patient comorbidity burden, stone characteristics, institutional ERCP/IOC availability, surgeon volume), which likely influence decision-making and could explain part of the observed practice variation. Seventh, preoperative abdominal ultrasound, used by 99% of respondents, has well-recognized limitations in patients with severe obesity due to suboptimal acoustic windows and operator-dependent sensitivity; this may result in underdiagnosis of gallstones or gallbladder pathology and should be considered when interpreting the high rate of ultrasound-based screening in this cohort. Eighth, the surveyed cohort was 99% male, reflecting the current gender distribution of bariatric surgeons in Egypt; this limits the generalizability of findings to female-predominant surgical environments and should be considered in any cross-national comparison. Finally, the study was not designed to assess patient outcomes; therefore, the results cannot establish comparative effectiveness of concomitant versus delayed strategies and should be interpreted as a description of current expert practice rather than evidence of superiority.

Future work should build directly on the willingness of surgeons to collaborate. A pragmatic next step is an Egyptian multicenter registry specifically tracking baseline gallstone status, bariatric procedure type, whether cholecystectomy was concomitant or staged, use of UDCA, IOC/imaging strategy in altered anatomy, and hard outcomes (operative time, bile duct injury, leaks, readmissions, and subsequent biliary interventions).

## Conclusion

Experienced Egyptian bariatric surgeons are consistent in screening and generally prefer concomitant cholecystectomy when gallstones are documented, while avoiding prophylactic excision of a normal gallbladder. Practice still varies meaningfully in UDCA use, drain management, antibiotic duration, and intraoperative biliary imaging, highlighting where standardization efforts should be prioritized. However, the results described here represent contemporary expert opinion and self-reported preferences; prospective, outcome-validated multicenter registry data are required before definitive conclusions on comparative effectiveness can be drawn or formal standardization recommended. The practical next step is to establish a shared national pathway informed by current consensus points and to collect multicenter outcomes data that reflect Egypt’s specific clinical reality, as a prerequisite for evidence-based national recommendations.

## Data Availability

No datasets were generated or analysed during the current study.

## Declarations

### Collaborators

The bariatric surgeons who participated in the survey are acknowledged as collaborators for their valuable contributions to data collection and for sharing their clinical practices and perspectives.
